# Retraction: Association of P16-RBSP3 inactivation with phosphorylated-RB1 over-expression in basal-parabasal layers of normal-cervix unchanged during CACX development

**DOI:** 10.1042/BCJ20160323_RET

**Published:** 2025-06-02

This article (“Association of P16-RBSP3 inactivation with phosphorylated RB1 overexpression in basal–parabasal layers of normal cervix unchanged during CACX development” DOI: 10.1042/BCJ20160323) is being retracted from the *Biochemical Journal* at the request of the Editor-in-Chief and the Editorial Board. This follows the receipt of a notification from a reader, alerting the Editorial Board to irregularities in the background details of Figures 1Bii, 3Ai, and 3Bi. Additionally, the Editorial Office has noted similarities between figures in two papers from this author group. The first noted is between this paper’s Supplementary Figure S2C p-RB1 x #6261 N panel and the Figure 1 HIF-1α #3678 N panel from Chakraborty et al, 2018 (DOI: 10.1042/BCJ20170649). The second is between this paper’s Supplementary Figure 3 RBSP3 x #3450 N panel and the Figure 1 VHL x #4554 N panel from Chakraborty et al, 2018 (DOI: 10.1042/BCJ20170649).

The authors were contacted regarding the concerns raised and provided original data for Figures 1Bii, 3Ai, and 3Bi, as well as the relevant histology images; however, these have not resolved the issues identified. The Editorial Board therefore stands by the decision to retract.

